# The Transcription Unit Architecture of *Streptomyces lividans* TK24

**DOI:** 10.3389/fmicb.2019.02074

**Published:** 2019-09-06

**Authors:** Yongjae Lee, Namil Lee, Yujin Jeong, Soonkyu Hwang, Woori Kim, Suhyung Cho, Bernhard O. Palsson, Byung-Kwan Cho

**Affiliations:** ^1^Systems and Synthetic Biology Laboratory, Department of Biological Sciences and KI for the BioCentury, Korea Advanced Institute of Science and Technology, Daejeon, South Korea; ^2^Systems Biology Research Group, Department of Bioengineering, University of California, San Diego, San Diego, CA, United States; ^3^Department of Pediatrics, University of California, San Diego, San Diego, CA, United States; ^4^Novo Nordisk Foundation Center for Biosustainability, Technical University of Denmark, Lyngby, Denmark; ^5^Intelligent Synthetic Biology Center, Daejeon, South Korea

**Keywords:** *Streptomyces*, transcription, transcription start site, transcription termination, transcription unit

## Abstract

*Streptomyces lividans* is an attractive host for production of heterologous proteins and secondary metabolites of other *Streptomyces* species. To fully harness the industrial potential of *S. lividans*, understanding its metabolism and genetic regulatory elements is essential. This study aimed to determine its transcription unit (TU) architecture and elucidate its diverse regulatory elements, including promoters, ribosome binding sites, 5′-untranslated regions, and transcription terminators. Total 1,978 transcription start sites and 1,640 transcript 3′-end positions were identified, which were integrated to determine 1,300 TUs, consistent with transcriptomic profiles. The conserved promoter sequences were found as 5′-TANNNT and 5′-TGAC, representing the −10 and −35 elements, respectively. Analysis of transcript 3′-end positions revealed the presence of distinctive terminator sequences and the RNA stem structure responsible for the determination of the 3′-boundary of a transcript. Functionally related genes are likely to be regulated simultaneously by using similar promoters and being transcribed as a poly-cistronic TU. Poly-cistronic TUs were further processed or alternatively transcribed into multiple TUs to fine-regulate individual genes in response to environmental conditions. The TU information and regulatory elements identified will serve as invaluable resources for understanding the complex regulatory mechanisms of *S. lividans* and to elevate its industrial potential.

## Introduction

A large group of Gram-positive filamentous bacteria, *Streptomyces*, possesses a long linear chromosome with high GC content (approximately 70%) and is characterized by complex life cycle, accompanied by morphological and physiological changes (Chater, 1989; Bentley et al., 2002; Flardh and Buttner, 2009). *Streptomyces* has been noticed for the ability to produce a wide range of antibiotics as the products of diverse and complex secondary metabolism (Demain, 1999; Berdy, 2005; Procopio et al., 2012). The secondary metabolites produced by *Streptomyces* include valuable bioactive compounds such as anthelmintic agents, anticancer agents and immunosuppressants, emphasizing the importance of the genus. Recent advances in next-generation sequencing (NGS) and computational tools revealed additional uncharacterized, biosynthetic gene clusters for novel secondary metabolites present in the genomes of *Streptomyces*, further elevating its industrial and clinical potential (Blin et al., 2017; Liu et al., 2018). In addition to their ability to produce bioactive secondary metabolites, *Streptomyces* species are of great interest as heterologous expression hosts for industrial production of important proteins due to their remarkable protein secretion ability (Hamed et al., 2018). In particular, *Streptomyces lividans*, a genetically close relative of the model organism *Streptomyces coelicolor* A3(2), is a prominent host for recombinant protein production, since *S. lividans* displays reduced extracellular proteolytic activity compared to other *Streptomyces* species (Nakashima et al., 2005; Anne et al., 2014). Owing to its genetic composition suitable for protein production and the availability of well-established genetic tools, successful applications to the production of recombinant proteins and secondary metabolites derived from other *Streptomyces* species have been described in *S. lividans* so far (Liu et al., 2016; Hamed et al., 2017; Pyeon et al., 2017; Novakova et al., 2018). Yet, the production yields are often low and require optimization for heterologous expression (Anne et al., 2014). To elevate and fully harness the potential of *S. lividans* as a heterologous expression host, understanding its complex genetic regulation and the discovery of the corresponding regulatory elements are essential. Despite the industrial importance of *S. lividans*, most genetic studies have been implemented in the model organism *S. coelicolor*. Though the genome of *S. coelicolor* is similar to *S. lividans*, clear phenotypic differences have been observed, suggesting the presence of distinct genetic regulations in the two *Streptomyces* species (Hopwood et al., 1983; Ruckert et al., 2015). In this study, we applied four types of NGS techniques, including dRNA-Seq, Term-Seq, RNA-Seq, and Ribo-Seq, to determine the transcription units (TUs) and corresponding genetic regulatory elements for the individual TUs of *S. lividans*, and, ultimately, aimed to elevate its industrial potential (Cho et al., 2009; Ingolia et al., 2009; Levin et al., 2010; Sharma et al., 2010; Dar et al., 2016).

## Materials and Methods

### Strain and Culture Condition

The mycelium of *S. lividans* TK24 was maintained in 25% glycerol at −80^°^C. Cells were cultured in 50 mL R5 − media with 8 g glass beads (3 ± 0.3 mm diameter) at 30^°^C. The composition of R5 − medium is as follow: 5.73 g TES (pH 7.2), 103 g sucrose, 10 g glucose, 5 g yeast extract, 10.12 g MgCl_2_⋅6H_2_O, 0.25 g K_2_SO_4_, 0.1 g casamino acids, 0.08 mg ZnCl_2_, 0.4 mg FeCl_3_⋅6H_2_O, 0.02 mg CuCl_2_⋅2H_2_O, 0.02 mg MnCl_2_⋅4H_2_O, 0.02 mg Na_2_B_4_O_7_⋅10H_2_O, and 0.02 mg (NH_4_)_6_Mo_7_O_24_⋅4H_2_O in 1L distilled water. Cell growth was determined with biological triplicates. For dRNA-Seq, Term-Seq, RNA-Seq and Ribo-Seq, cultures were prepared as biological duplicates and sampled at 9.5, 14, 16, and 20 h after inoculation for early, mid- and late-exponential, and stationary phases, respectively. For Ribo-Seq, cultures were treated with thiostrepton for 5 min before harvesting the cells.

### RNA-Seq Library Preparation

After harvesting, the cells were washed with 500 μL of polysome buffer (20 mM Tris-HCl pH 7.5, 140 mM NaCl, 5 mM MgCl_2_), and resuspended with 500 μL of lysis buffer (0.3 M sodium acetate pH 5.2, 10 mM EDTA, 1% Triton X-100). The cell suspension was frozen with liquid nitrogen, and the frozen suspension was physically lysed by grinding using mortar and pestle. The lysate was centrifuged at 4^°^C for 10 min at 16000 × *g* and the supernatant was saved and stored at −80^°^C until used for RNA extraction. For RNA extraction, 100 μL of the supernatant was used. The volume of the supernatant was adjusted to 350 μL with DEPC-treated water and mixed with equal volume of phenol:chloroform:isoamyl alcohol = 25:24:1 solution. The mixture was then centrifuged and RNA was extracted from the upper aqueous phase with ethanol precipitation. To remove any DNA contamination, the RNA samples were treated with DNase I (New England Biolabs, Ipswich, MA, United States). To remove ribosomal RNA (rRNA), Ribo-Zero rRNA Removal Kit Bacteria (Epicentre, Madison, WI, United States) was used following the manufacturer’s instructions. The rRNA-depleted RNAs were visualized with 2% agarose gel electrophoresis for quality control. RNA-Seq libraries were constructed using TruSeq Stranded mRNA Library Prep Kit (Illumina, San Diego, CA, United States), following the manufacturer’s instructions.

### dRNA-Seq Library Preparation

Approximately 2.5 μg of DNase I treated RNA from the 4 sampling time points were mixed, and the rRNA in the RNA mixture was depleted using Ribo-Zero rRNA Removal Kit Bacteria (Epicentre). Approximately 700 ng of rRNA-depleted RNA was incubated in 1 × RNA 5′ polyphosphatase (TAP; Epicentre) reaction buffer and 1 U of SUPERase-In (Invitrogen, Carlsbad, CA, United States), the RNase inhibitor, with [TAP(+)] or without [TAP(−)] TAP at 37^°^C for 1 h. The reaction was cleaned up with ethanol precipitation and then 5 pmol of 5′ RNA adaptor (5′-ACACUCUUUCCCUACACGACGCUCUUCCGAUCU-3′) was ligated to the purified RNA with T4 RNA ligase (Thermo Fisher Scientific, Waltham, MA, United States) in 1 × RNA ligase buffer and 0.1 mg/mL BSA by incubating at 37^°^C for 90 min. The adaptor-RNA ligate was then purified using Agencourt AMPure XP beads (Beckman Coulter, Brea, CA, United States) according to the manufacturer’s instructions. The purified product was reverse-transcribed with SuperScript III Reverse Transcriptase (Invitrogen) and purified using Agencourt AMPure XP beads. The purified cDNA was divided in half and amplified and indexed using Phusion High-Fidelity DNA Polymerase (Thermo Fisher Scientific) for Illumina sequencing. The amplification step was first monitored using a CFX96 Real-Time PCR Detection System (Bio-Rad Laboratories, Hercules, CA, United States) with SYBR Green I Nucleic Acid Gel Stain (Invitrogen). The remaining half of the cDNA was amplified and stopped 1 cycle before the signal becomes totally plateau. Finally, the amplified library was purified using Agencourt AMPure XP beads, and the concentration of the library was measured with Qubit 2.0 fluorometer (Invitrogen). The size distribution of the library was checked by gel electrophoresis on 2% agarose gel.

### Ribo-Seq Library Preparation

Before harvesting, cells were pre-treated for 5 min with thiostrepton to inhibit translation elongation. The harvested cells were washed with polysome buffer (20 mM Tris-HCl pH 7.4, 140 mM NaCl, 5 mM MgCl_2_, and 33.5 μg/mL thiostrepton) and resuspended with lysis buffer (475 μL Polysome buffer, 25 μL Triton X-100, and 6 μL DNase I). The cell suspension was frozen with liquid nitrogen and lysed by grinding using mortar and pestle. The cell lysate was centrifuged at 4^°^C for 10 min at 16,000 × *g* and the soluble supernatant was recovered. Ribosome-unprotected RNA was digested by incubating with RNase I (Invitrogen) at 37^°^C for 45 min. After RNase I digestion, RNase reaction was inactivated by treatment with SUPERase-In and monosomes were recovered using a Sephacryl S-400 column (GE Healthcare, Chicago, IL, United States). Ribosome protected RNA fragments were recovered using phenol:chloroform:isoamyl alcohol = 25:24:1 solution and rRNA was depleted with Ribo-Zero rRNA Removal Kit Bacteria (Epicentre) according to the manufacturer’s instructions. After rRNA depletion, RNA was resolved on a 15% TBE-urea gel and 26–34 nt RNA fragments were size-selected. The size-selected RNAs were eluted in 300 mM sodium acetate pH 5.2, 1 mM EDTA and 0.25% SDS. The eluate was ethanol precipitated and libraries were constructed with NEB Next small RNA library prep set (New England Biolabs) according to the manufacturer’s instructions. The constructed libraries were divided in half and amplified and indexed using Phusion High-Fidelity DNA Polymerase for Illumina sequencing. The amplification step was first monitored on a CFX96 Real-Time PCR Detection System (Bio-Rad Laboratories) with SYBR Green I Nucleic Acid Gel Stain (Invitrogen). The remaining half of the library was amplified and stopped 1 cycle before the signal becomes totally plateau. The amplified libraries were further size-selected on 2% agarose gel with MinElute Gel Extraction Kit (Qiagen, Hilden, Germany).

### Term-Seq Library Preparation

Term-Seq libraries were constructed as previously described with some modifications (Dar et al., 2016). 1.25 μg of DNase I-treated RNA from 4 time points were mixed and treated with Ribo-Zero rRNA Removal Kit Bacteria (Epicentre) to deplete rRNA prior to adaptor ligation. Further, 500–900 ng of rRNA-depleted RNA was mixed with 1 μL of 150 μM amino-blocked DNA adaptor (5′-p-NNAGATCGGAAGAGCGTCGTGT-3′), 2.5 μL of 10 × T4 RNA ligase 1 buffer, 2.5 μL of 10 mM ATP, 2 μL of DMSO, 9.5 μL of 50% PEG8000, and 2.5 μL of T4 RNA ligase 1 (New England BioLabs). The mixture was incubated at 23^°^C for 2.5 h, purified with Agencourt AMPure XP beads (Beckman Coulter) and eluted with 9 μL DEPC-treated water. The RNA-adaptor ligates were then fragmented using fragmentation buffer (Ambion, Inc., Austin, TX, United States) by incubating at 72^°^C for 90 s. After fragmentation, the products were purified with Agencourt AMPure XP beads and eluted with 8 μL DEPC-treated water. The fragmented RNA was reverse transcribed using 1 μL of 10 μM reverse transcription primer (5′-TCTACACTCTTTCCCTACACGACGCTCTTC-3′) with SuperScript III Reverse Transcriptase (Invitrogen) according to the manufacturer’s instructions. After reverse transcription, the cDNA was purified with Agencourt AMPure XP beads and eluted with 5 μL DEPC-treated water. The purified cDNA was subjected to another adaptor ligation cycle as above, with increased incubation time (8 h) and different amino-blocked adaptor sequence (5′-p-NNAGATCGGAAGAGCACACGTCTGAACTCCAGTCAC-3′). After adaptor ligation, the product was purified using Agencourt AMPure XP beads and indexed by PCR for 10 cycles with Phusion High-Fidelity DNA Polymerase using forward (5′-AATGATACGGCGACCACCGAGATCTACACTCTTTCCCTA CACGACGCTCT-3′) and reverse (5′-CAAGCAGAAGACG GCATACGAGATNNNNNN (6 nt index) GTGACTGGAGTT CAGAC-3′) primers.

### High-Throughput Sequencing

All libraries were sequenced using Illumina HiSeq 2500 platform with either 1 × 100 bp (RNA-Seq and dRNA-Seq) or 1 × 50 bp (Ribo-Seq and Term-Seq) read length. The reads were trimmed and mapped to the *S. lividans* TK24 genome (Accession number CP009124). For RNA-Seq and Term-Seq, reads were reversely mapped to reference.

### Identification of Transcription Start Sites (TSSs)

TSSs were identified as previously described (Jeong et al., 2016). The 5′-end position of dRNA-Seq reads from the TAP(+) library were considered to be potential TSSs. Briefly, the potential TSSs that were less than 100 bp apart from the ones located at neighboring positions were clustered together. The potential TSSs adjacent to other potential TSSs in the same cluster were then sub-clustered together based on the standard deviation of their genomic positions (<10). Only potential TSS clusters with more than three read counts were considered and the potential TSSs with maximum read counts within each sub-cluster were selected as TSSs. The read counts of selected TSS positions from the TAP(+) and TAP(−) libraries were then compared and positions with more read counts in the TAP(−) library were discarded. Further, the selected TSSs were manually inspected using the corresponding RNA-Seq profile (Jeong et al., 2016).

### Identification of 3′-End Positions of RNA Transcripts

Each 3′-end position of Term-seq read indicates the *in vivo* address of 3′-end position of each RNA transcript, resulting from transcription termination or post-transcriptional processing including programed mRNA cleavage and RNA decay. Only the 3′-end position of Term-Seq reads located within intergenic regions including up to 10 bp downstream of the gene were considered as potential transcript 3′-end positions (TEPs). The positions were clustered together based on the distance from adjacent positions (<10 bp). Within each cluster, the read count of each position was assumed to follow normal distribution and read count enriched positions were deduced by calculating the modified *z*-score as previously described with some modifications (Lalanne et al., 2018).

$$Z\,\left(x\right)=\frac{r\,\left(x\right)-\mu \,\left(r\,\left(x\right)\right)}{\sigma \,\left(x\right)}$$

where

$$\mu \,\left(r\,\left(x\right)\right)=\frac{1}{N\,\left(x\right)-1}\,\left(-r\,\left(x\right)+\sum_{y\in C\,\left(x\right)}r\,\left(y\right)\right),$$

$$\sigma \,\left(x\right)=\sqrt{\mu \,\left(r\,{\left(x\right)}^{2}\right)-\mu \,{\left(r\,\left(x\right)\right)}^{2}}$$

*Z*(*x*) is the modified *z*-score at position*x*, *r*(*x*) is the read count of evaluated position *x*. μ(*r*(*x*))and σ(*x*) are the mean and standard deviation of read counts of other positions in the cluster except the evaluated position, respectively. *N*(*x*) is the length of the cluster containing position *x* and *C*(*x*) is the set of positions within the cluster containing position *x*.

The positions with read counts of less than 3 or modified *z*-scores less than 3 were discarded. Among the remaining potential TEPs, the reproducible positions with the highest read count within the intersecting region of clusters from two biological replicates were selected as TEPs. For example, if genomic positions from 3 to 25 were clustered together for one replicate and genomic positions from 13 to 42 were clustered together for another replicate, the potential TEP with the highest read count within the genomic positions from 13 to 25 was selected as the TEP.

### Motif Discovery

The sequence elements of promoters were identified with the MEME suite (Bailey et al., 2009). The sequences between 20 bp upstream and 1 bp downstream of each TSS were utilized to identify − 10 elements, and the sequences between 40 bp and 25 bp upstream of each TSS were utilized to identify − 35 elements. The − 10 element was found by using the MEME suite with zoops (Zero or One Occurrence Per Sequence) option. The − 35 element was found with oops (One Occurrence Per Sequence) option and only the sequences, whose *P*-value was less than 0.05, were regarded as the motif. After finding each sequence element, 21 nt sequences of −10 elements (5′-N_8_TANNNTN_7_) and 16 nt sequences of −35 elements (5′-N_5_TGACN_7_) were extracted and the sequence motifs were written by using Weblogo (Crooks et al., 2004). Spacer length was calculated when the two promoter elements were found for one TSS. For the promoter motif of *S. coelicolor*’ *hrdB* regulons, *hrdB* target genes and their primary TSSs were collected from published ChIP-Seq and dRNA-Seq data (Jeong et al., 2016; Smidova et al., 2019). The promoter motifs of *S. lividans* were found by similar methods using the MEME suite oops option. The ribosome binding site (RBS) motif was found by using the method for −35 element detection with up to 25 nt 5′-UTR sequences upstream of start codons of genes, whose 5′-UTR is longer than 10 nt. The −10 element sequence upstream of start codons of leaderless genes was found by the same method for RBS detection with 25 nt sequences upstream of start codons. For terminator sequence analysis, sequences from 41 bp upstream to 20 bp downstream of each TEP were collected and used for sequence alignment and motif discovery, and upstream 41 bp sequences were used for ΔG prediction. The sequence alignment and motif were created using Weblogo, and ΔG was predicted using RNAfold with temperature parameter of 30^°^C (Lorenz et al., 2011).

### Detection of TUs

Briefly, adjacent TSSs and TEPs were paired together for determination of the TUs. In case of *cis*-regulatory TEPs, they were allowed to form TU only with TSSs assigned to the same gene. To capture the poly-cistronic TUs, the maximum intergenic distance between two adjacent genes was assumed as 500 bp. For primary, secondary and internal TSSs, any combination of TSSs and TEPs was allowed to form TU unless every intergenic distance in the TU did not exceed 500 bp. For antisense and intergenic TSS, 1 kbp downstream region was scanned for the presence of the TEP or start codon of a gene. TU was then determined if a TEP was present in that region. If the start codon of a gene appeared in that region, TUs were determined by the same method as the primary, secondary or internal TSSs. The determined TUs were then compared to the RNA-Seq profile, informing the removal of false-positives. Any potential TUs supported by TSS, TEP, and RNA-Seq profiles but not detected from computational processes were manually inspected. The determined TUs were then categorized into mono-cistronic or poly-cistronic TUs based on the number of associated genes. For TUs starting from internal TSS, the TSS assigned gene was not considered as “associated.” TUs lacking associated genes were classified as either *cis*-regulatory or sRNA based on the distance from TSS to start position of the downstream gene (<500 bp).

## Results and Discussion

### Genome-Wide Identification of TSSs

**FIGURE 1:**
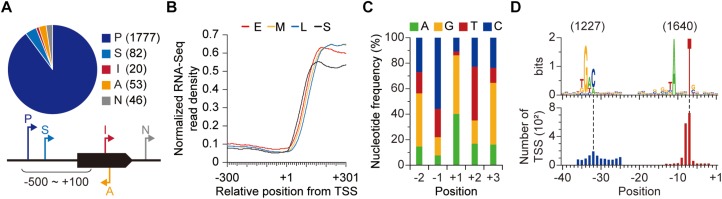
Genome-wide identification of transcription start sites (TSSs). **(A)** TSSs identified from dRNA-Seq. **(B)** RNA-Seq read density across the identified TSSs. **(C)** Nucleotide frequency near the TSSs. **(D)** The conserved promoter sequence of *S. lividans* TK24. Each promoter motif was discovered separately.

The TSS is an important genomic location as it provides a starting point for the elucidation of regulation of gene expression. By exploiting differential RNA-Seq (dRNA-Seq) approach, we experimentally identified TSSs of *S. lividans* TK24 genome. Since *Streptomyces* display polymorphous growth and dynamic gene expression, we sampled at four different growth phases (early-, mid- and late-exponential, and stationary phases) to determine TSSs (Supplementary Figure 1; Jeong et al., 2016). In principle, the presence of triphosphate at the 5′-end of bacterial primary transcript can be utilized to distinguish TSSs from the monophosphorylated or hydroxylated 5′-end of processed transcript (Soutourina et al., 2013). From the dRNA-Seq results (see the materials and methods for sequencing statistics), 1,978 TSSs were determined and classified into five categories based on their genomic positions to nearby genes (Figure 1 and Supplementary Dataset 1; Jeong et al., 2016). First, TSS located near the start position of an annotated gene (from 500 nt upstream to 100 nt downstream of the start position of the gene) was considered responsible for the transcription of the gene and classified as either primary (P) or secondary (S) TSS based on the read counts. Total 1,777 and 82 TSSs were assigned to primary and secondary TSSs, respectively. Among the unclassified TSSs, 20 and 53 TSSs located within a gene and in the reverse strand of a gene were classified as internal (I) and antisense (A) TSSs, respectively. Lastly, the remaining 46 TSSs were categorized as intergenic (N) TSSs. Such diversity of genomic positions and the number of TSSs in each category reflect complexity of genetic regulation, suggesting the presence of novel transcripts encoded by the *S. lividans* genome. To confirm whether the determined TSSs are genuine or not, we measured the whole transcriptome for all the time points using RNA-Seq (Supplementary Table 1; Ingolia et al., 2009; Levin et al., 2010). The transcriptome data showed great correlation for biological duplicates of a specific growth phase but differences were observed between different growth phases, which reflects the dynamic gene expression of the strain (Supplementary Figure 2; Jeong et al., 2016). The RNA-Seq read density increased in accordance with the TSS positions, which strongly supports that the determined TSSs are genuine (Figure 1B).

### Determination of Regulatory Elements in Transcription Initiation

The genomic position of TSS leads to information on regulatory elements for transcription initiation. Thus, we analyzed the sequence elements affecting transcription initiation.

First, we examined the preference for specific nucleotides near the TSS. Any of the four nucleotides (A, G, T, and C) can be utilized for TSS (+1 site), but in most cases (more than 85%), transcription starts from either A or G, which are purines (Figure 1C). On the contrary, C and T, which are pyrimidines, were dominant for −1 and +2 sites, respectively. Especially, the dominance of T for +2 site is unusual, considering the high GC-content (more than 70%) of *Streptomyces* genomes. The alternate occurrence of purines at template strand and non-template strand across the TSS is consistent with the beneficial effect of base stacking interaction for binding of the first and second nucleotides (Basu et al., 2014).

Second, we examined the promoter upstream from the TSS. The promoter sequence is recognized by RNA polymerase and its associated σ factor, and it determines the location for the onset of transcription (Browning and Busby, 2004). From the identified TSSs, conserved sequence elements of promoters were detected to be 5′-TANNNT and 5′-TGAC for − 10 and − 35 elements of the promoter, respectively, using the MEME suite (Figure 1D; Bailey et al., 2009). Promoter recognition by RNA polymerase holoenzyme is a fundamental transcriptional regulatory mechanism and the affinity of σ factor to a specific promoter is dependent on promoter sequence. *Streptomyces* typically encodes a remarkable number of σ factors in the genome and 62 σ factors are present in the *S. lividans*’ genome (Staron et al., 2009; Ruckert et al., 2015; Rebets et al., 2018).

**FIGURE 2:**
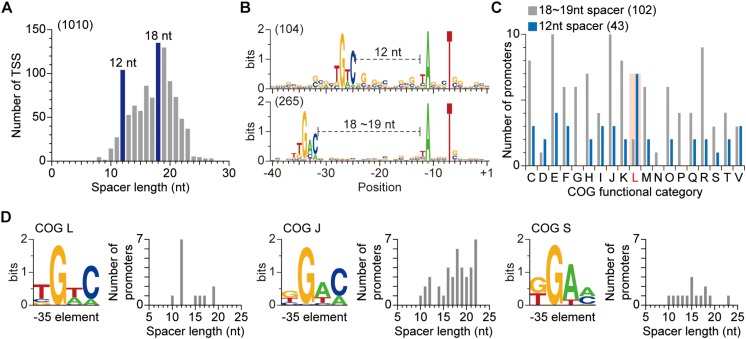
Relationship between promoter sequences and the function of the corresponding gene. **(A)** The spacer lengths of *S. lividans* TK24. The spacer length was calculated for transcription start sites whose −10 and −35 elements were simultaneously discovered. **(B)** Differences in promoter sequences with different spacer length. **(C)** Functional analysis of genes exploiting two different types of promoter. Genes with 12 nt spacer are functionally enriched in COG category L, which represents DNA replication, recombination and repair. **(D)** Variation of −35 element sequence and spacer length dependent on the function of genes.

Third, we examined the spacer between the promoter elements. From the 1,010 TSSs whose −10 and −35 promoter elements were simultaneously identified, the spacers between two promoter elements were collected. The spacers varied in length, from 8 to 27 nt, reflecting the huge number of σ factors in the *S. lividans*’ genome. Most promoters had 18- or 19-nt spacers (Figure 2A). Unexpectedly, there was also enrichment for 12-nt spacer. The promoters with the 12-nt spacer utilized slightly different −35 element sequence (TGTC) compared to the promoters with 18- or 19-nt spacer (TGAC) (Figure 2B). The variation in −35 element sequence and spacer length suggest that different σ factors are involved in the regulation of genes exploiting the two groups of promoters. Since σ factors play a key role in the interplay between environmental signals and cellular responses, we analyzed the differences in function of genes regulated by the promoters with different spacer lengths based on Clusters of Orthologous Groups (COG) assignment (Supplementary Table 2; Tatusov et al., 2000; Wu et al., 2011). Genes exploiting promoters with 18- or 19-nt spacer were broadly distributed to diverse COG categories especially to category C, E, J, K, or R (Figure 2C).

Fourth, we sought to associate a σ factor use with the identified promoters. From the functional analysis, we hypothesized that genes exploiting promoters with 18- or 19-nt spacer are regulated by housekeeping σ factors, as their enriched functions are related to fundamental processes, including cellular resource metabolisms, translation and transcription. Based on the published ChIP-Seq and dRNA-Seq results in the genetically close organism *S. coelicolor*, we examined this hypothesis (Jeong et al., 2016; Smidova et al., 2019). The binding specificity of *S. lividans*’ *hrdB*, a housekeeping σ factor, was assumed to be the same as the *S. coelicolor*’ *hrdB* since their amino acid sequences are identical. The TSSs of the regulons of *S. coelicolor*’ *hrdB* were collected and their promoter motifs were analyzed. The sequence of each promoter element was quite comparable to that of the total TSSs of *S. lividans* (Supplementary Figure 3A). In accordance with our hypothesis, the spacer lengths of promoters of *S. coelicolor*’ *hrdB* regulon were enriched to 18- or 19-nt and no peak for 12-nt was observed (Supplementary Figure 3B). However, genes exploiting promoters with 12-nt spacer, independent of *hrdB*, were functionally enriched in COG category L, which represents DNA replication, recombination and repair (Figure 2C). Among the TSS identified σ factors, two σ factors (SLIV_13900 and SLIV_16385) were found to exploit 12-nt spacers for their promoters. Homologs for both σ factors are present in *S. coelicolor* (SCO4895 and SCO4409, respectively) and especially, the promoter sequence of SCO4895 is identical to SLIV_13900 (Bentley et al., 2002; Ruckert et al., 2015; Jeong et al., 2016). A transcriptomic study of *S. coelicolor* revealed that expression of SCO4895 coincides with DNA repair related genes when exposed to ciprofloxacin, suggesting that SLIV_13900 is the putative regulator of DNA repair related genes (Patkari and Mehra, 2013). Taken together, the variation of promoters, especially in their −35 sequences and spacer lengths, is important for orthogonal regulation of functionally distinct genes by specific σ factors.

Finally, in order to further examine the relationship between the function of a gene and selection of corresponding promoter, the −35 element and spacer length of genes in each COG category were analyzed. Not only the genes in category L, but also the genes in category F, J, N, S, and V exploited other sequences as the −35 element, rather than the widely conserved TGAC (Figure 2D and Supplementary Figure 4). Moreover, their spacer length distributions were different to that of other genes, for example, genes in category J had GGAG as the −35 element and the spacer length was enriched to 22 nt. Taken together, the variation in −35 element sequence and spacer length of promoters of functionally distinct genes indicate that the position and sequence of the −35 element play a key role for σ factor recognition, and also suggested that the genes in the regulons of a σ factor are functionally related to each other.

### Determination of 5′-Untranslated Regions

**FIGURE 3:**
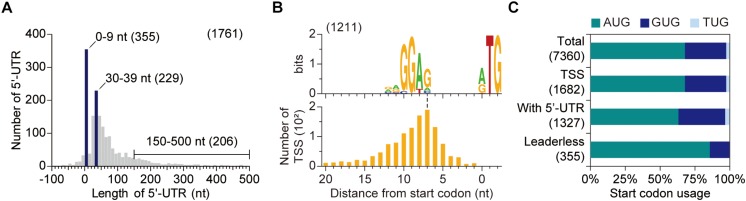
Determination of 5′-UTR. **(A)** The distribution of 5′-UTR length. Only primary transcription start sites assigned to coding DNA sequences were considered. **(B)** Conserved ribosome binding site (RBS) sequences of *S. lividans*. RBS sequence was discovered using up to 25 nt sequences upstream of start codon when the 5′-UTR is longer than 10 nt. **(C)** Start codon preference dependent on 5′-UTR length. AUG is preferred as a start codon for leaderless mRNA.

Important genetic information is found in the 5′-untranslated region (5′-UTR), that is the sequence between the TSSs and the start codon. We thus identified the 5′-UTR for of each gene. 5′-UTR contains a key regulatory sequence for translation and, in addition, unique structures in the 5′-UTR can mediate co-transcriptional or post-transcriptional regulation under specific conditions (Shine and Dalgarno, 1974; Dar et al., 2016; Sherwood and Henkin, 2016). The lengths of 5′-UTRs were broadly distributed (Figure 3A). To our surprise, approximately 20% of open reading frames (ORFs) were transcribed as the form of leaderless mRNA (mRNA with 5′-UTR of 0–9 nt in length), and more than 90% of them had no 5′-UTR (i.e., 5′-UTR of 0 nt) which indicates that transcription starts from the start codon of the corresponding ORF. The presence of leaderless transcripts is also observed in *S. coelicolor* with similar proportion (21%) (Jeong et al., 2016). The lack of 5′-UTR may hamper gene expression since 5′-UTR generally contains an RBS, which is partially complementary to 16S rRNAs and guides ribosome to align at the start codon of the downstream ORF (Shine and Dalgarno, 1974).

The sequence analysis of leadered mRNAs (mRNAs with 5′-UTRs of longer than 10 nt) revealed the presence of a conserved sequence, 5′-RRGGAG, upstream of the start codon which acts as the RBS of *S. lividans* (Figure 3B). However, promoter motif, rather than RBS motif, was found upstream of leaderless genes (Supplementary Figure 5). The lack of RBS for leaderless genes may hamper translation initiation and thus another strategy is required for efficient expression of leaderless genes. As a consequence, AUG was dominantly utilized as a start codon for ORFs in leaderless mRNAs, compared to total ORFs or ORFs in leadered mRNAs (Figure 3C). The AUG preference as the start codon of ORFs in leaderless mRNA indicates that strong interaction between anticodon of initiator tRNA and start codon is important for translation initiation of ORFs in leaderless mRNAs (Beck and Moll, 2018).

### Comparison of Translation Efficiency Between Leadered and Leaderless mRNAs

The transcription levels of certain genes often do not coincide with their actual abundance as fully functional proteins and one major cause is the presence of translational regulation (de Sousa Abreu et al., 2009; Ning et al., 2012). Regulations during translation dominantly take place during translation initiation and 5′-UTR is the most responsible element for translation initiation (Salis et al., 2009). To reveal the effect of start codon and 5′-UTR on translation, we conducted Ribo-Seq experiments, which capture ribosome-bound RNA fragments and measure gene expression at translational level (Supplementary Table 3 and Supplementary Figures 6A,B; Ingolia et al., 2009). From the transcriptome and translatome data, the translation efficiency (TE) of a gene was calculated as the Reads Per Kilobase of transcript, per Million mapped reads (RPKM) of the gene based on Ribo-Seq divided the RPKM of the gene based on RNA-Seq to evaluate the effect of start codon on translation (Supplementary Dataset 2). TE can serve as a measure for the translational performance of a transcript and can partly account for the imbalance between transcriptional expression and protein abundance.

We first evaluated the start codon dependence of the TE. For both leaderless and leadered mRNAs, TE was higher for genes exploiting AUG start codon, compared to other start codons, supporting the hypothesis that selection of start codon affects translation (Supplementary Figure 7A). The expression of leaderless mRNAs was generally lower than the expression of leadered mRNAs. The low expression of leaderless mRNAs may demand for higher TE and, in turn, AUG start codon (Supplementary Figure 7A). Next, we evaluated the presence of RBS on TE. Among 1,327 genes, whose 5′-UTR is longer than 10 nt, 1,211 genes were found to have RBS sequences upstream of start codon (Figure 3B). The TE values of 116 genes lacking RBSs upstream of their start codons were generally lower compared to genes with RBSs (Supplementary Figure 7B). However, the differences in TE values at late-exponential phase and stationary phase were not significant and, perhaps, the resource limited condition at late growth phase may alter the dependence of translation efficiency on sequence elements.

### Comparison of the General Regulatory Elements Between *S. lividans* and *S. coelicolor*

*Streptomyces lividans* is genetically close to the model actinomycete, *S. coelicolor*, however, the two organisms differ in their metabolism (Millan-Oropeza et al., 2017). To analyze the underlying regulatory elements that give rise to the differences between *S. coelicolor* and *S. lividans*, we compared the regulatory elements found near TSSs of the two organisms by using the previously published data from our group (Jeong et al., 2016). We compared the sequence elements of the TSSs, and it was found that the nucleotide frequency near the TSSs are similar between the two organisms (Supplementary Figure 8A). In addition, the conserved promoter sequence elements, such as −35 element and −10 element, of the two organisms were quite comparable to each other in terms of the sequences and positions (Supplementary Figure 8B). It is noteworthy that the amino acid sequences of the housekeeping sigma factor, *hrdB*, of the two organisms are identical and, therefore, their corresponding recognition motif should be similar.

Next, we compared the length of the 5′-UTR. The distributions of 5′-UTR lengths were similar for the two organisms and both strains utilized considerable number of leaderless genes (20.16% and 20.92% for *S. lividans* and *S. coelicolor*, respectively), whose 5′-UTR length is shorter than 10 nt (Supplementary Figure 8C). The 5′-UTR contains a key regulatory sequence for translation, RBS, and both organisms have similar purine-rich RBS in their 5′-UTR (Supplementary Figure 8D). Among the TSS identified genes, 1,282 gene pairs are well conserved between the two organisms (length difference <20%, amino acid similarity> 80%). We compared their 5′-UTR lengths, and found that 683 gene pairs (53.28%) have exactly same length of 5′-UTR and differences of 5′-UTR lengths of 867 gene pairs (67.63%) are less than 10 nt (Supplementary Figure 8E). This suggest that many of the homologs may undergo similar transcriptional and translational control in *S. coelicolor* and *S. lividans*.

Next, we compared the differences in secondary metabolism between *S. coelicolor* and *S. lividans*. Among the 29 biosynthetic gene clusters (BGCs) in *S. coelicolor*’ genome, 27 BGCs are well conserved in *S. lividans* (Nett et al., 2009). We analyzed the differences in temporal regulation of 27 conserved BGCs based on DESeq2 normalization (Love et al., 2014). Overall, the expression changes of BGCs across the growth are similar in both organisms. However, we observed that 6 gene homologs in 3 BGCs were differentially regulated for the two organisms (expression foldchange more than 2, *P*-value < 0.05) (Supplementary Figure 9A). For coelichelin BGC, 3 genes of *S. coelicolor* (SCO0489, SCO0492 and SCO0498) were down-regulated during late-exponential phase, while their homologs of *S. lividans* (SLIV_35495, SLIV_35480 and SLIV_35450) were up-regulated. Similarly, SCO7221 of germicidin BGC was down-regulated during mid-exponential phase, while *S. lividans*’ homolog, SLIV_03190, was up-regulated. Meanwhile, the overall gene expression pattern of each BGC is synchronous, suggesting that the master regulator for each BGC is differentially expressed in the two organisms. Interestingly, two gene homologs in actinorhodin BGC were differentially regulated during stationary phase, while other genes in the actinorhodin BGC were up-regulated in both *S. coelicolor* and *S. lividans*. It is noteworthy that the two differentially regulated gene homologs, SLIV_12965 and SLIV_12970 of *S. lividans* and SCO5084 and SCO5083 of *S. coelicolor*, are related to the transport of actinorhodin. Inactivation of the actinorhodin transporter leads to decrease in actinorhodin production and the differential regulation of actinorhodin transporter during stationary phase in the two organisms may lead to difference in the actinorhodin production of for the two organisms (Xu et al., 2012). Indeed, SLIV_12965 and SLIV_12970 was down-regulated during stationary phase and actinorhodin production of *S. lividans* was low in our culture condition (estimated by the color of media). Then we analyzed the underlying genetic regulatory element that leads to differential expression of the transporter genes. There are about 80 single nucleotide variances (SNVs) and a 12 nt deletion in the 21 kbp actinorhodin BGC. Interestingly, 15 SNVs are enriched in the 137 bp intergenic region between SLIV_12970 and SLIV_12975, where the promoter of SLIV_12970 is located (TSS for SLIV_12970 was not detected, and the TSS for SLIV_12970 was approximated by utilizing the TSS information of SCO5083, which is the homolog of SLIV_12970) (Supplementary Figure 9B). Among them, 6 SNVs are within 5′-UTR and 9 SNVs are upstream of TSS. The SNVs upstream of TSS may alter the transcription level of the transporters in *S. lividans*, leading to lower production of actinorhodin in *S. lividans*.

### Genome-Wide Identification of Transcript 3′-End Positions

**FIGURE 4:**
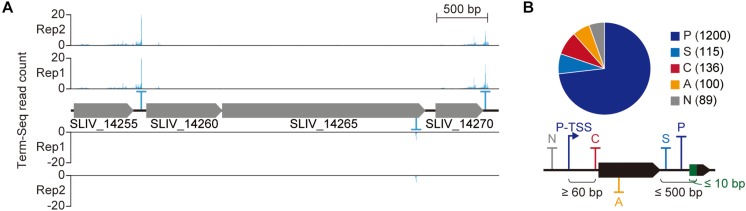
Genome-wide identification of transcript 3′-end positions. **(A)** Example of Term-Seq 3′-end profile. **(B)** Transcript 3′-end positions identified from Term-Seq.

Though the precise functional role of 3′-termini of bacterial transcripts remains poorly understood compared to the 5′-termini, transcript 3′-termini affects gene expression in various manner (Miyakoshi et al., 2015; Dar et al., 2016; Ren et al., 2017). To map the positions where transcripts end, we exploited Term-Seq (see the materials and methods for sequencing statistics). In contrast to dRNA-Seq, the sequencing adaptors are ligated to the 3′-termini of transcripts and, as a result, the transcripts’ 3′-ends are sequenced. From the Term-Seq results, a total of 1,640 transcript 3′-end positions (TEP) were identified across the genome (Figure 4A and Supplementary Dataset 3). Since Term-Seq lacks a control library that enables distinguishing transcripts from either transcription termination or post-transcriptional processing, each TEP where Term-Seq reads are enriched indicates the 3′-boundary of a TU. Similar to the TSS classification, the determined TEPs were classified into five categories to examine their functional roles to nearby genes (Figure 4B). A TEP located less than 500 nt from an upstream gene was considered to be responsible for separating the expression of the upstream gene from the downstream region and classified as either primary (P) or secondary (S) TEP based on the Term-Seq read counts. A total of 1,200 and 115 TEPs were assigned to primary and secondary TEPs, respectively. On the contrary, 89 TEPs located more than 500 nt from upstream genes were classified as intergenic (N) TEPs. A TEP located in the reverse strand of a gene was classified as antisense (A) TEP. If a TEP was located between the downstream gene and its corresponding primary TSS, the TEP was classified as *cis*-regulatory (C) TEP. A total of 136 TEPs were assigned to this category. It is noteworthy that the minimum length between a primary TSS and *cis*-regulatory TEP was set as 60 nt for proper transcription termination. Again, the diversity of genomic positions of TEPs and the number of TEPs in each category suggest the presence of complex regulatory mechanisms for gene expression.

### Determination of Regulatory Elements in Transcription Termination

**FIGURE 5:**
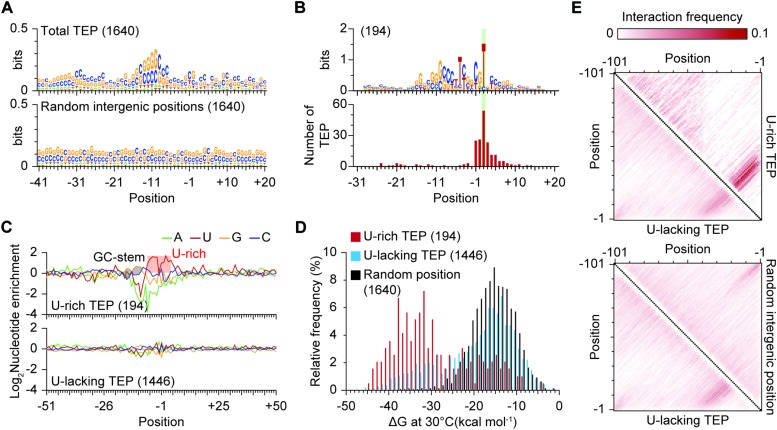
Identification of a U-rich motif near transcript 3′-end positions (TEPs). **(A)** Sequence alignment near TEPs or randomly selected intergenic positions. The 41 nt upstream to 20nt downstream sequences of TEPs were used for sequence logo generation using Weblogo. **(B)** The conserved U-rich motif. **(C)** Nucleotide enrichment analysis of the two types of TEPs. Nucleotide enrichment was calculated by dividing the frequency of each nucleotide for the TEP sets with the frequency of the same nucleotide of randomly selected intergenic positions. **(D)** ΔG distribution of TEPs. The ΔG was calculated from upstream 41 nt sequences, including TEPs or randomly selected intergenic positions using RNA fold with the temperature parameter of 30^°^C. **(E)** Structure comparison between U-lacking TEPs, U-rich TEPs and randomly selected intergenic positions.

To elucidate the sequence determinant for the 3′-boundary of transcripts, we analyzed the nucleotide sequences near TEPs. The sequence alignment showed that higher occurrence of GC-rich nucleotides is present upstream of the TEPs compared to randomly selected intergenic positions (Figure 5A). In bacteria, GC-rich RNA stem followed by a U-rich tail functions as a transcription terminator independently of transcription termination factor, Rho (Gusarov and Nudler, 1999). A sequence motif that contains GC-rich region followed by a U-rich region was identified in relatively small number of TEPs (194 of 1,640), which may act as a Rho-independent transcription terminator in *S. lividans* (Figure 5B). Comparing to known Rho-independent terminators of other bacteria, the enrichment of U was relatively weak in the TEPs (U-rich TEP) (Dar et al., 2016). However, considering the fact that the frequency of G and C is extremely high in *Streptomyces* (>70%), the frequency of U is higher than that observed in randomly selected intergenic positions. This supports the hypothesis that U-rich TEPs are the product of Rho-independent transcription termination (Figure 5C).

In the sequence alignment of all TEPs, GC-rich region was found upstream of TEPs and may form a stable RNA stem structure by the strong interaction between G and C. The RNA stem structure formation is critical for determination of the 3′-end of a transcript for both Rho-dependent and Rho-independent transcription termination (Dar and Sorek, 2018). For Rho-dependent transcription termination, the RNA stem structure serves as a protectant from RNase activity (Dar and Sorek, 2018). To examine whether the upstream GC-rich sequences stabilize the RNA secondary structures near TEPs, which may induce transcription termination or protect mRNA from nuclease cleavage, local RNA structures near TEPs were predicted using RNAfold (Lorenz et al., 2011). The ΔG value of the predicted RNA structure was considered as a parameter that indicates the stability of RNA. The ΔG values of local RNA structure of TEPs were relatively lower than those of random positions, which indicates that local RNA structures of TEPs are more stable than those of randomly selected positions (Figure 5D). Surprisingly, the ΔG values of U-rich TEPs were relatively lower than those of TEPs without a U-rich motif (U-lacking TEP). Moreover, the U-lacking TEPs showed similar distribution in the ΔG values of RNA structures to randomly selected positions, suggesting that those TEPs may lack RNA stem structures. To test whether both U-rich and U-lacking TEPs are genuine TEPs or not, RNA-Seq profile across the TEPs were examined (Supplementary Figure 10). The RNA-Seq read density sharply decreased in accordance with the TEPs, indicating that both U-rich and U-lacking TEPs are *bona fide*. However, the U-lacking TEPs showed less changes in read density compared to U-rich TEPs, suggesting that RNA polymerase often continues to transcribe mRNA after the U-lacking TEPs (read-through effect).

Yet, the underlying features that determine U-lacking TEPs as genuine TEPs are ambiguous. The GC-rich nature of the *Streptomyces* genomes may result in low ΔG values for randomly selected positions, and thus we analyzed the RNA structure itself, rather than the ΔG value of RNA that indirectly represents RNA structure. RNA structure was predicted for 101 nucleotides upstream of TEPs or randomly selected positions and the frequency of base interactions between the two positions was calculated. The base interactions were enriched around 20 nt upstream of TEPs for both U-rich and U-lacking TEPs (Figure 5E). However, such enrichment of base interactions was not observed in randomly selected positions. The randomly selected regions from the GC-rich *Streptomyces* genome contain naturally plenty of G and C and strong interactions between G and C are frequently formed across the transcripts. ΔG values of the predicted RNA structure from either U-lacking TEPs or randomly selected positions are similar to each other. But the difference lies in the presence of RNA stem structure upstream of the position. Overall, formation of RNA stem structure is a critical determinant for the 3′-boundary of a transcript.

### High-Throughput Detection of TSSs and TEPs Leads to Determination of TUs

**FIGURE 6:**
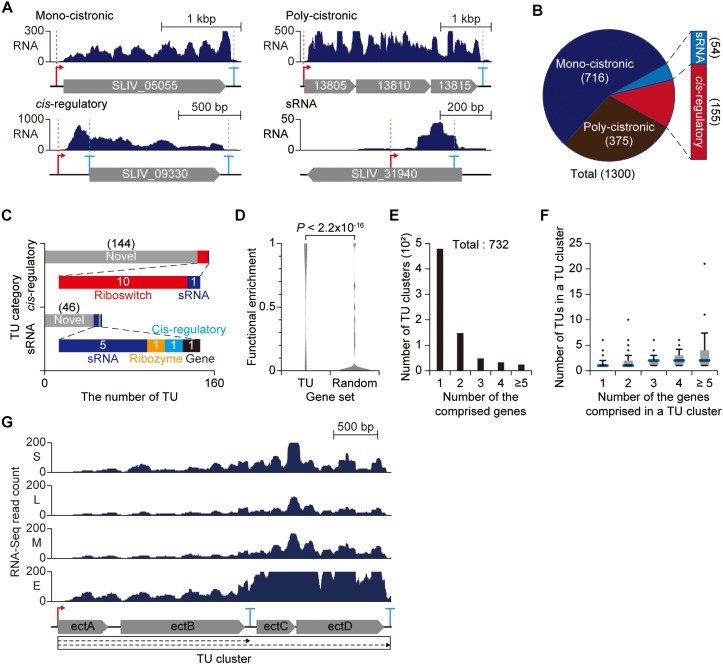
Genome-wide determination of transcription units (TUs). **(A)** Example of each TU category. **(B)** The number of TUs determined from transcription start site (TSS) and transcript 3′-end position (TEP) information. **(C)** Characterization of non-coding TUs. sRNA and *cis*-regulatory TUs were searched against Rfam database. **(D)** The functional relatedness of genes in a same poly-cistronic TU. The functional enrichment was determined as the maximum frequency of genes with same COG functional category in a single poly-cistronic TU. **(E)** The number of TU clusters separated by the number of comprised genes. **(F)** Proportional relationship between the number of TUs in a TU cluster and the number of genes in the TU cluster. **(G)** The RNA-Seq profile of TU cluster for ectoine biosynthesis genes.

The high-throughput detection of TSSs and TEPs led to determination of 1,300 TUs encoded in the *S. lividans* genome (Supplementary Dataset 4). The TUs were categorized into four categories based on the number of genes encoded by the TU and the relative position of TSS to the corresponding downstream genes (Figures 6A,B). TUs encoding only one gene were classified as mono-cistronic TUs, and TUs encoding more than one gene were assigned to poly-cistronic TUs. A TU lacking any encoding gene was classified as either sRNA or *cis*-regulatory TU based on the distance between TSS and downstream genes, which may encode unannotated genes or act as regulatory elements. To understand their functions comprehensively, the sRNA and *cis*-regulatory TUs were analyzed by Rfam, resulting in 144 TUs for *cis*-regulatory and 46 TUs for sRNA functions (Kalvari et al., 2018). The remaining TUs were determined as riboswitches, sRNAs, ribozymes, *cis*-regulatory elements and genes (Figure 6C).

A total of 375 TUs were found to be transcribed as poly-cistronic transcripts. The poly-cistronic transcripts are advantageous to rapid and simultaneous modulation of multiple genes encoded in the same transcript. To harness the advantage of poly-cistronic structure, it is obvious that genes in the same poly-cistronic TU are functionally related (*e.g*., secondary metabolite biosynthetic gene clusters). COG analysis showed that the genes located in the same poly-cistronic TU were functionally related to each other with great significance compared to randomly chosen gene sets (*p*-value < 2.2 × 10^–16^) (Figure 6D). Together with the promoter sequence and gene function analysis, such integrity in the functions of the genes in poly-cistronic TUs can serve as an efficient genomic structure for the organism to fully operate with limited number of σ factors.

However, employing poly-cistronic TU structure is undesirable for fine-tuning of individual gene in response to changes in environmental conditions. Interestingly, *S. lividans* genome encodes overlapped TUs sharing one or more genes within the TUs, suggesting the presence of complex regulation coordinated by the alternative use of TSSs or TEPs (Cho et al., 2009). The TU variants sharing genes provide alternative regulatory modes under different environmental conditions and thus enable subtle and precise modulation of genes transcribed in poly-cistronic mRNA. To examine the interconnected landscape of TUs, mono-cistronic or poly-cistronic TUs that share at least one gene or indirectly connected by another TU were clustered together and defined as TU clusters (Mao et al., 2015).

A total of 732 TU clusters were determined where most genes were transcribed independent of other genes and the number of genes comprising a TU cluster was generally proportional to the number of TUs comprising the same TU cluster (Figures 6E,F). The increasing number of TUs in TU clusters with multiple genes may be derived from alternative use of promoters and terminators or post-transcriptional processing. For example, the TU cluster for ectoine biosynthesis genes (*ectA*, *ectB*, *ectC*, and *ectD*) is composed of 2 TUs (Figure 6G). One TEP was detected at the intergenic region between *ectB* and *ectC*. Surprisingly, 5′-ends of transcripts were extensively mapped to the intergenic region (both TAP(+) and TAP(−) libraries) (Supplementary Figure 11). The presence of a TEP and enriched transcript 5′-ends at the intergenic region suggests the presence of endonucleolytic cleavage to dissect a TU into two TUs (in this case, the TU encoding *ectC* and *ectD* was not determined since the 5′-end present in the intergenic region between *ectB* and *ectC* was not determined as a TSS). And the corresponding RNA-Seq profile changes across the intergenic region, resulting in biased expression of *ectC* and *ectD* from *ectA* and *ectB*. The inconsistent expression across the ectoine biosynthetic TU cluster is highly likely due to different stability between the transcript bearing *ectA* and *ectB* and the transcript bearing *ectC* and *ectD*. Taken together, such complex TU architecture defined by a set of TSS and TEP serves as an efficient strategy to fine-tune the gene expression and balance the stoichiometry of individual gene in response to diverse environmental conditions.

## Conclusion

*Streptomyces* have complex life cycle and undergo drastic morphological and physiological changes with the transition from primary to secondary metabolism (Chater, 1989; Flardh and Buttner, 2009; Jeong et al., 2016). Changes in transcriptome and translatome are responsible for the complex transition. To fully utilize the industrial potential of *Streptomyces*, understanding the regulatory mechanisms for transcription and translation are essential. In this study, we exploited high-throughput sequencing techniques and identified 1,978 TSSs and 1,640 TEPs of *S. lividans* TK24 genome at a nucleotide resolution. By integrating these data, 1,300 TUs were determined, which are interconnected and regulated by diverse regulatory elements for transcription and translation.

From the TSS information, diverse *cis*-regulatory sequences affecting gene expression were examined. For TSS and +2 position, unusual enrichment of A and T was observed, respectively. The promoter, which is recognized by RNA polymerase complex and is responsible for the transcription initiation, was detected from 1,978 TSSs and the conserved sequences of −10 and −35 elements were found as 5′-TANNNT and 5′-TGAC, respectively. The sequence of −35 element and spacer length were diverse and the selection of −35 element and spacer of promoter was relevant to the function of the corresponding genes. *Streptomyces* encode approximately 60 σ factors in their genomes, which are far more than other bacteria (Staron et al., 2009). The function of σ factor is to recognize a specific promoter sequence and recruit RNA polymerase for transcription initiation (Browning and Busby, 2004). The relationship between the function of a gene and its corresponding promoter suggests that the genes in a σ factor regulon are functionally related and the number of σ factors reflects the wide range of phenotypic spectrum in *Streptomyces*. In addition to the *cis*-regulatory sequences responsible for transcription initiation, a *cis*-regulatory sequence affecting translation initiation was identified in the 5′-UTR, which is defined by the TSS and start codon of the corresponding gene. The RBS, which is highly complementary to the 3′-terminus of 16S rRNA and guides ribosome to properly align at the start codon, was found as 5′-RRGGAG (Shine and Dalgarno, 1974). Interestingly, approximately 20% of genes were transcribed as leaderless mRNAs with a high preference of AUG as a start codon, suggesting the interaction between start codon and initiator tRNA is important for translation initiation of genes without 5′-UTR (Beck and Moll, 2018).

In bacteria, transcription is terminated mostly by either Rho-independent or Rho-dependent termination mechanism, and Rho-independent transcription termination is prevalent (Ray-Soni et al., 2016). The Rho-independent terminator is typically composed of GC-rich stem structure followed by U-rich track (Gusarov and Nudler, 1999). Analysis of 1,640 TEPs revealed the presence of a distinct U-rich terminator motif, which may act as Rho-independent terminator in *S. lividans* (Figure 5B). The other TEPs without a U-rich motif were less structured compared to the U-rich TEPs and comparable to randomly selected genomic positions evaluated by the ΔG from the predicted RNA structure (Figure 5D). However, structural analysis revealed the presence of stem structure directly upstream of TEPs and the formation of stem structure is a key determinant for the TEPs (Figure 5E). The TEPs from Rho-dependent transcription termination also possess RNA stem structure upstream of them, suggesting that the U-lacking TEPs are generated from Rho-dependent transcription termination (Dar and Sorek, 2018).

In conclusion, the TU architecture revealed the presence of novel potential regulatory elements, including sRNA and *cis*-regulatory TUs. In addition, genes encoding in a poly-cistronic TU were functionally related to each other, enabling simultaneous regulation of multiple genes involved in a specific function. Moreover, the functional relatedness of genes in a poly-cistronic TU is analogous to the functional relatedness of genes under a specific promoter, suggesting that gene expression is regulated as a functional unit. Our study resolved the complex TU architecture for poly-cistronic TUs and provided evidence suggesting that a poly-cistronic TU is further processed into multiple TUs to fine-regulate the stoichiometry of each gene in the TU. The identified regulatory elements of individual TUs will broaden our understanding of complex regulatory mechanisms of *Streptomyces* and lead to full utilization of the potential of *S. lividans* as a heterologous expression host for industrial enzymes and secondary metabolites.

## Data Availability

The datasets, RNA-Seq, Term-Seq, RNA-Seq, and Ribo-Seq, generated for this study have been deposited in European Nucleotide Archive (ENA) under accession number of PRJEB31507.

## Author Contributions

B-KC designed the study. YL, YJ, NL, SH, and WK performed the experiments. YL, SC, and B-KC performed the data analysis. YL, SC, BP, and B-KC wrote the manuscript.

## Conflict of Interest Statement

The authors declare that the research was conducted in the absence of any commercial or financial relationships that could be construed as a potential conflict of interest.
