# Supplementary material for: The Transcription Unit Architecture of Streptomyces lividans TK24
Source: Front Microbiol. 2019 Sep 6;10:2074. doi: 10.3389/fmicb.2019.02074 (PMC6742748; doi:10.3389/fmicb.2019.02074)
Supplement: Supplementary file 1 [file Data_Sheet_1.PDF]

Supplementary Figures

1.1 Supplementary Figures

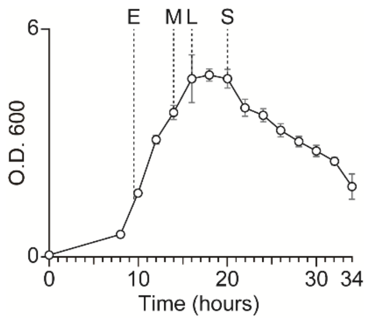

Supplementary Figure 1. Growth profile of *S. lividans* TK24 in R5− medium.

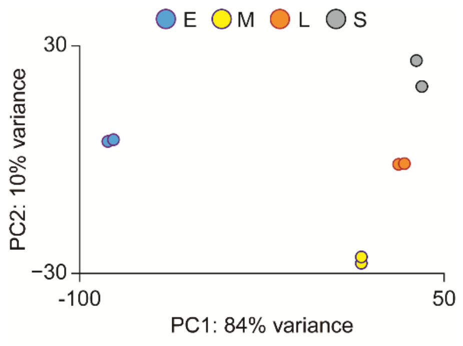

Supplementary Figure 2. PCA analysis based on RNA-Seq.

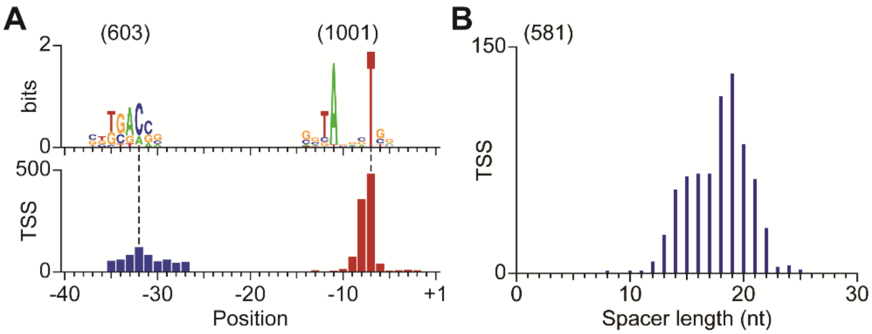

Supplementary Figure 3. Putative promoter sequence recognized by *S. coelicolor*' *hrdB*. **(A)** The promoter motif found in regulons of *S. coelicolor*' *hrdB*. **(B)** The spacer length distribution.

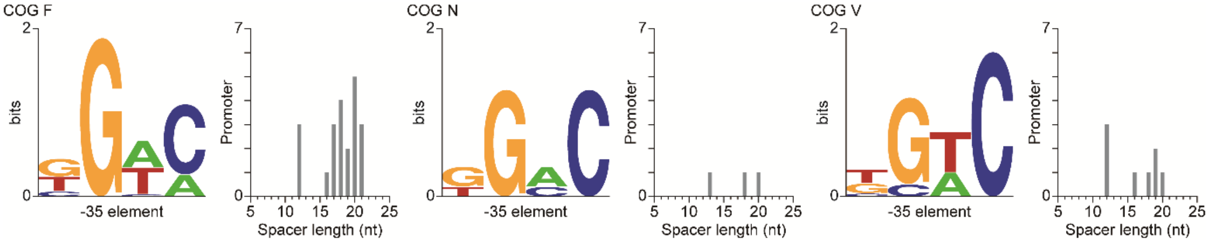

Supplementary Figure 4. Variation of –35 element sequence and spacer length dependent on the function of genes.

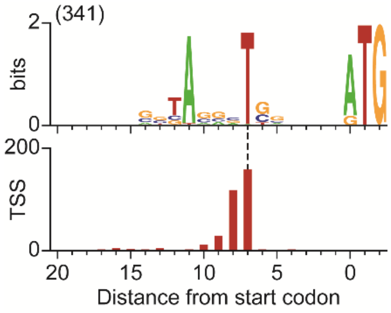

Supplementary Figure 5. **–**10 element sequence found upstream of start codon of leaderless genes.

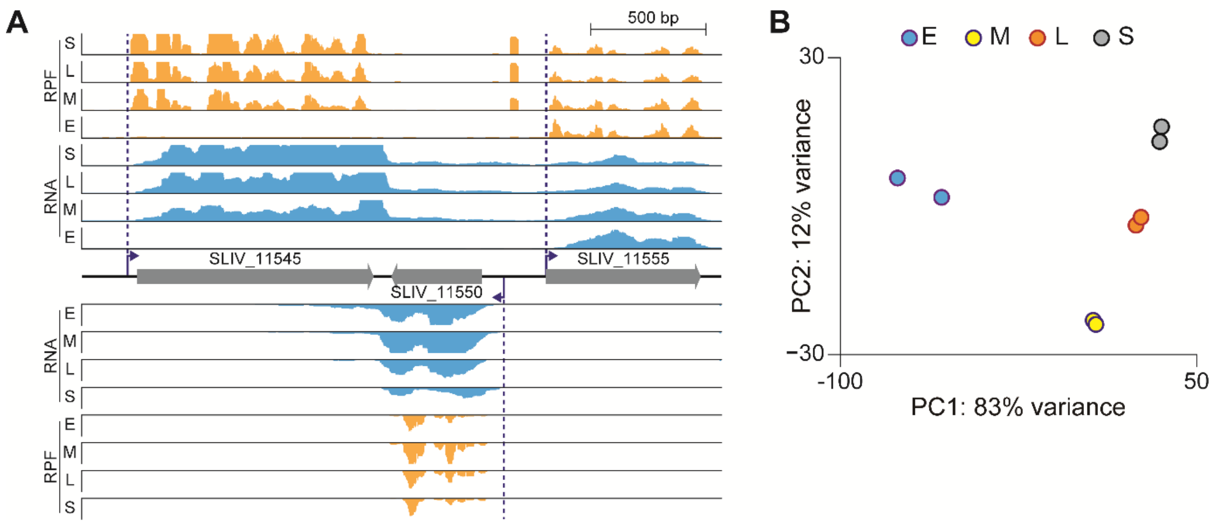

Supplementary Figure 6. Ribo-Seq results. **(A)** Example of determined TSSs and corresponding RNA-Seq and Ribo-Seq profile. **(B)** PCA analysis based on Ribo-Seq.

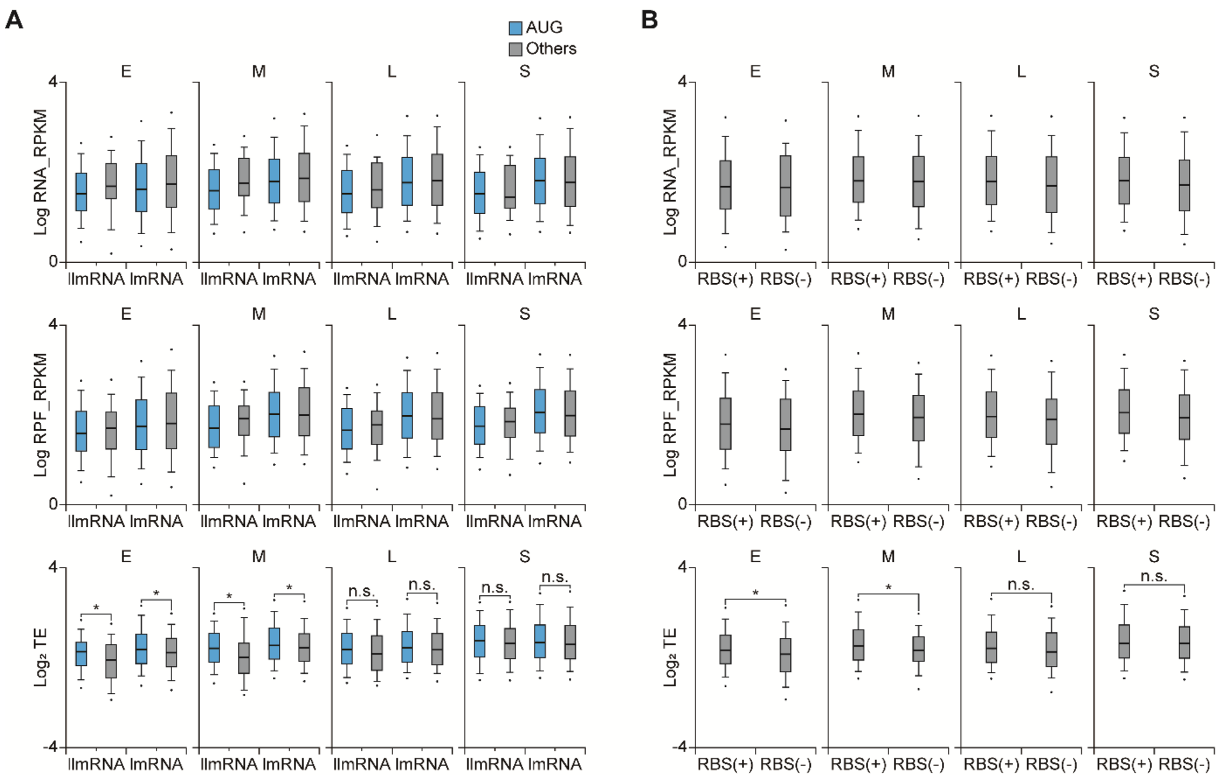

Supplementary Figure 7. Comparison of expression and TE between genes exploiting different regulatory elements. **(A)** Gene expression and TE of leaderless and leadered mRNAs. llmRNA and lmRNA stand for leaderless mRNA and leadered mRNA, respectively. * stands for P-value < 0.05. **(B)** Expression and TE of genes with or without RBS sequence. * stands for P-value < 0.05.

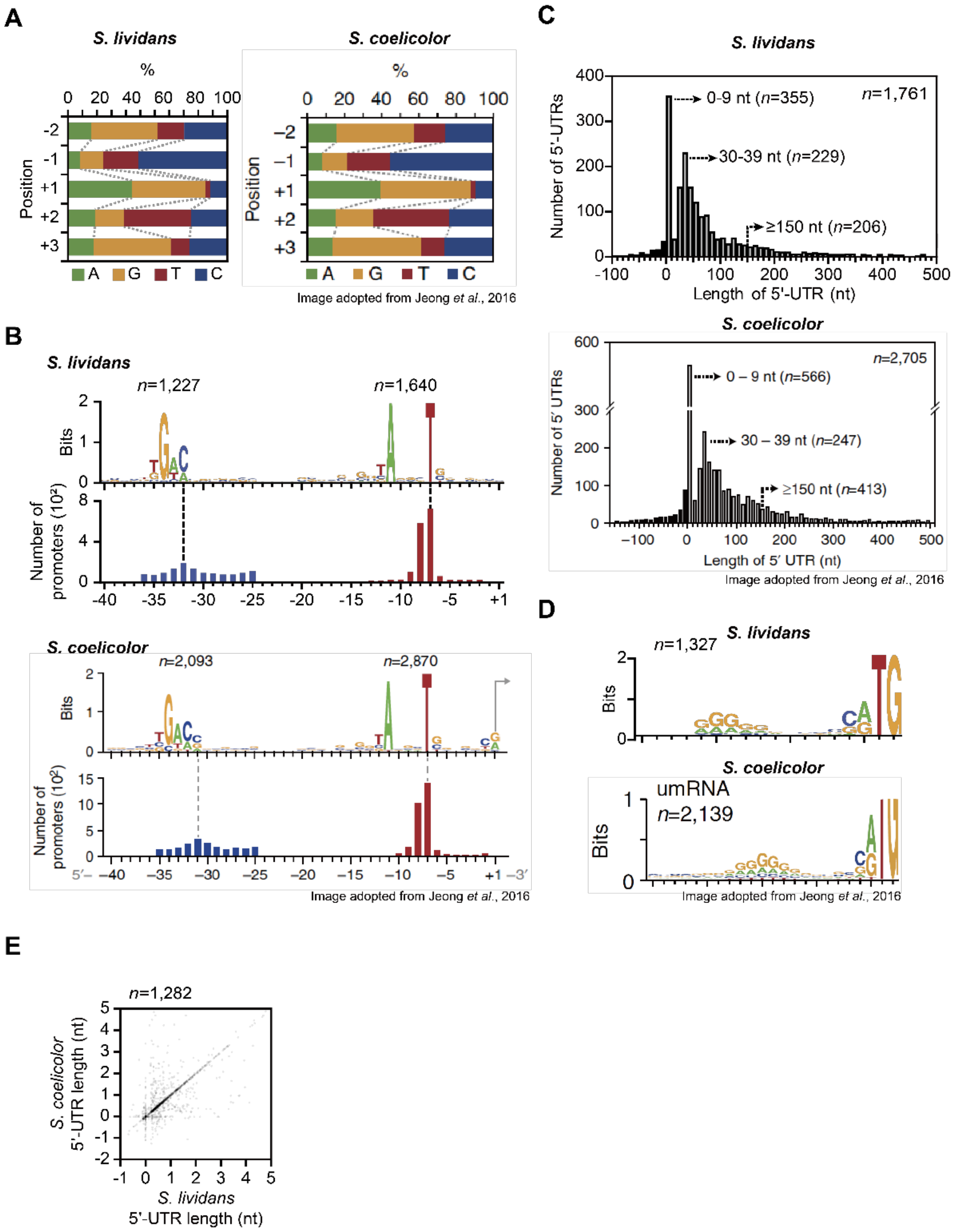

Supplementary Figure 8. Comparison of regulatory elements between *S. coelicolor* and *S. lividans*. **(A)** Nucleotide frequency near the TSSs. **(B)** The conserved promoter sequences of *S. lividans* and *S. coelicolor*. **(C)** The distribution of 5′-UTR lengths of *S. lividans* and *S. coelicolor*. **(D)** Conserved RBS sequences of *S. lividans* and *S. coelicolor* found in leadered mRNAs. Motif was discovered by using the method of Jeong *et al*. **(E)** Comparison of 5′-UTR lengths of homologous genes in *S. coelicolor* and *S. lividans*. The 5′-UTR was determined from the primary TSS. For *S. coelicolor*, image was adopted from previous study (Jeong et al., 2016).

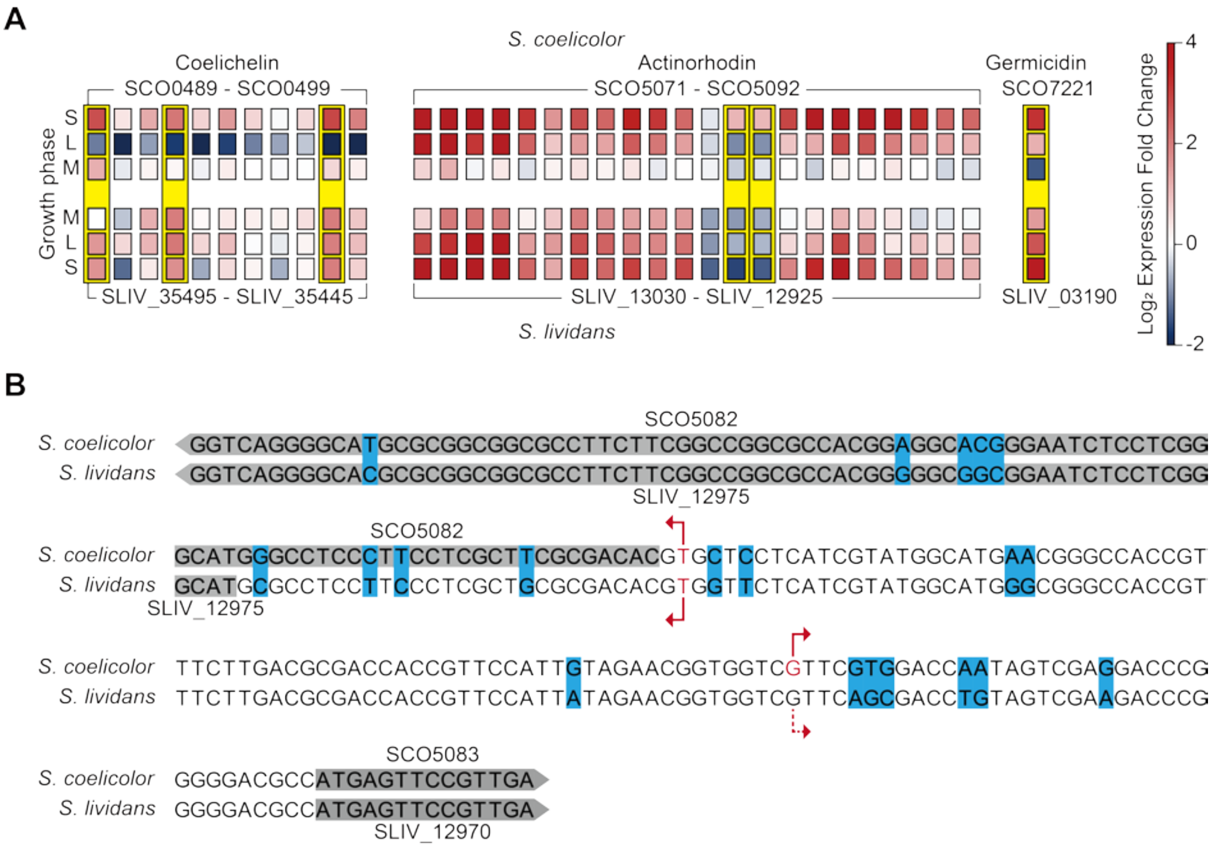

Supplementary Figure 9. Differential expression of BGCs in *S. coelicolor* and *S. lividans*. **(A)** Expression pattern of BGCs containing differentially regulated genes. Differentially expressed genes (more than 2 fold expression change, *P*-value 0.05 in both *S. coelicolor* and *S. lividans*) are denoted as yellow. Gene expression fold change value relative to early-exponential phase was used. **(B)** Differences in nucleotide sequences between *S. coelicolor* and *S. lividans* in the promoter region of actinorhodin transporter genes. Red arrow indicates detected transcription start site. Dotted red arrow indicates the predicted transcription start site of SLIV_12970. Mismatches in the sequences are denoted as blue.

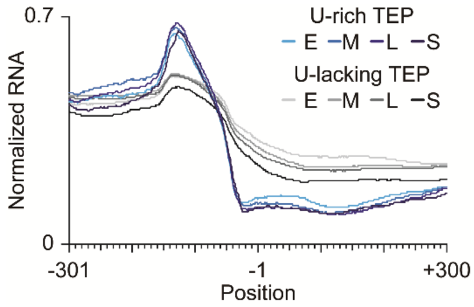

Supplementary Figure 10. RNA-Seq read density across the identified TEPs.

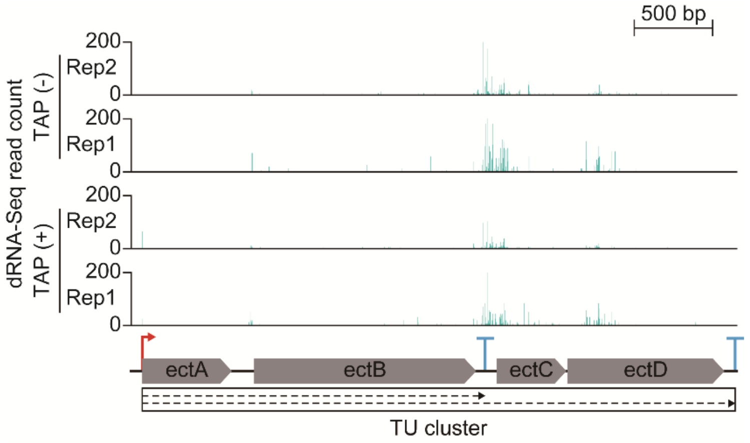

Supplementary Figure 11. The dRNA-Seq 5′-end profile of TU cluster for ectoine biosynthesis genes.

References

Jeong, Y., Kim, J.N., Kim, M.W., Bucca, G., Cho, S., Yoon, Y.J., et al. (2016). The dynamic transcriptional and translational landscape of the model antibiotic producer *Streptomyces coelicolor* A3(2). *Nat Commun* 7**,** 11605. doi: 10.1038/ncomms11605.
